# Vital Signs: Current Cigarette Smoking Among Adults Aged ≥18 Years with Mental Illness — United States, 2009–2011

**Published:** 2013-02-08

**Authors:** Joe Gfroerer, Shanta R. Dube, Brian A. King, Bridgette E. Garrett, Stephen Babb, Timothy McAfee

**Affiliations:** Center for Behavioral Health Statistics and Quality, Substance Abuse and Mental Health Services Administration; Office on Smoking and Health, National Center for Chronic Disease Prevention and Health Promotion, CDC

## Abstract

**Background:**

Cigarette smoking remains the leading cause of preventable morbidity and mortality in the United States. Despite overall declines in cigarette smoking, a high prevalence of smoking persists among certain subpopulations, including persons with mental illness.

**Methods:**

Combined data from the 2009–2011 National Survey on Drug Use and Health (NSDUH) were used to calculate national and state estimates of cigarette smoking among adults aged ≥18 years who had any mental illness (AMI), defined as having a mental, behavioral, or emotional disorder, excluding developmental and substance use disorders, in the past 12 months.

**Results:**

During 2009–2011, an annual average of 19.9% of adults aged ≥18 years had AMI; among these persons, 36.1% were current smokers, compared with 21.4 % among adults with no mental illness. Smoking prevalence among those with AMI was highest among men, adults aged <45 years, and those living below the poverty level; smoking prevalence was lowest among college graduates. During 2009–2011, adults with AMI smoked 30.9% of all cigarettes smoked by adults. By U.S. region, smoking prevalence among those with AMI was lowest in the West (31.5%) and Northeast (34.7%) and highest in the Midwest (39.1%) and South (37.8%), with state prevalence ranging from 18.2% (Utah) to 48.7% (West Virginia).

**Conclusions:**

The prevalence of cigarette smoking is high among adults with AMI, especially for younger adults, those with low levels of education, and those living below the poverty level; the prevalence varies by U.S. region.

**Implications for Public Health Practice:**

Increased awareness about the high prevalence of cigarette smoking among persons with mental illness is needed to enhance efforts to reduce smoking in this population. Proven population-based prevention strategies should be extended to persons with mental illness, including implementing tobacco-free campus policies in mental health facilities. Primary care and mental health-care providers should routinely screen patients for tobacco use and offer evidence-based cessation treatments. Given that persons with mental illness are at risk for multiple adverse behavioral and health outcomes, tobacco cessation will have substantial benefits, including a reduction in excess morbidity and mortality attributed to tobacco use.

## Introduction

Tobacco use remains the leading cause of preventable morbidity and mortality in the United States. The health consequences of tobacco use include cardiovascular disease, multiple types of cancer, pulmonary disease, adverse reproductive outcomes, and the exacerbation of chronic health conditions (1). Cigarette smoking causes approximately 443,000 premature deaths in the United States annually and has been estimated to cost the United States $96 billion in direct medical expenses and $97 billion in lost productivity each year.*

Despite overall declines in the prevalence of adult cigarette smoking, prevalence remains high among certain subpopulations, particularly persons with mental illness (1). Research suggests that smoking prevalence among U.S. adults with mental illness or serious psychological distress ranges from 34.3% (phobias or fears) to 88% (schizophrenia), compared with 18.3% among adults with no such illness (2,3). Persons with mental illness might smoke more frequently and heavily than the general population (2), and they might lack access to cessation services (4,5). Monitoring tobacco use across all sub-populations† is necessary to meet the *Healthy People 2020* target of reducing the prevalence of cigarette smoking among adults to ≤12% (objective TU-1).§ Using data from the 2009–2011 National Survey on Drug Use and Health (NSDUH), this report provides the most recent national and state estimates of cigarette smoking among adults aged ≥18 years with AMI.

## Methods

NSDUH collects information on substance use and mental health indicators from a nationally representative sample of civilian, noninstitutionalized persons aged ≥12 years in the United States. Data are collected annually through handheld computer–assisted face-to-face interviews at the respondent’s residence, using a combination of interviewer-administered and respondent self-administered questions.¶ This study included 138,000 adult respondents interviewed during 2009, 2010, or 2011. Annual response rates ranged from 87% to 89% at the household level and from 74% to 76% at the individual level. To assess AMI in the preceding year, respondents aged ≥18 years answered a series of 14 questions that made up two scales measuring psychological distress (Kessler-6) and disability (World Health Organization Disability Assessment Schedule) (6). Kessler-6 assesses psychological distress and includes questions about feeling nervous, hopeless, restless or fidgety, sad or depressed, or worthless (6). The World Health Organization Disability Assessment Schedule assesses disturbances in social adjustment and behavior, including psychological difficulties that interfere with respondents remembering, concentrating, getting out on their own, participating in familiar and unfamiliar social activities, and taking care of daily responsibilities related to home, work, or school (6). Scores on these two scales were used to determine AMI status based on a statistical model developed from clinical interviews that assessed disorders based on criteria in the Diagnostic and Statistical Manual of Mental Disorders (DSM-IV)¶; these clinical interviews were administered to a nationally representative subsample of NSDUH respondents. AMI was defined as having a mental, behavioral, or emotional disorder, and did not include developmental and substance use disorders, in the past 12 months.

Current smoking was defined as smoking all or part of a cigarette within the 30 days preceding the interview. Among current smokers, daily smoking was defined as smoking every day in the past 30 days. Ever smoking was defined as adults who smoked at least 100 cigarettes in their lifetimes. The quit ratio was calculated as the percentage of adults who had ever smoked ≥100 cigarettes and who also reported no past month cigarette use. Current smoking was examined by age, race/ethnicity, education, poverty status, U.S. Census region, and year (https://www.census.gov/hhes/www/poverty/data/threshld/index.html), both overall and by sex. Persons identified as Hispanic might be of any race. Persons identified as white, black, Asian, American Indian/Alaska Native, or Other were all non-Hispanic. The six racial/ethnic categories were mutually exclusive. Other included Native Hawaiians or Other Pacific Islanders and persons of two or more races. Poverty status was defined using poverty thresholds published by the U.S. Census Bureau. Data were weighted during analysis to adjust for the differential probability of both selection and response. Statistical significance of observed differences was assessed using chi-square tests of independence between subgroups, and pairwise tests for specific comparisons of interest. A level of 0.05 was used to determine statistical significance.

## Results

During 2009–2011, an estimated annual average of 19.9% (45.7 million) U.S. adults aged ≥18 years had AMI (Table 1). The prevalence of current smoking was 36.1% among persons with AMI and 21.4% among those without AMI (Table 2). The prevalence of adult smokers aged ≥18 years with AMI was 29.5%. Among current smokers, the average number of cigarettes smoked in the preceding month was higher among adults with AMI (331 cigarettes) compared with adults who did not have AMI (310) (p<.05).** Among all cigarettes smoked by adults aged ≥18 years, 30.9% were smoked by adults with AMI.** Among adults with AMI, the quit ratio was 34.7%, compared with 53.4% among adults who did not have AMI (p<0.05).

Prevalence of current smoking among adults with AMI was higher among men (39.6%) than women (33.8%) (Table 2). By age, prevalence was higher for those aged 18–24 years (41.6%) and 25–44 years (40.5%) than for those aged 45–64 years (33.5%) and ≥65 years (13.0%). By race/ethnicity, prevalence was lowest among Asians (20.6%) and highest among whites (37.7%) and respondents categorized as of Other race (40.0%). However, the difference between smoking prevalence among Asians with AMI compared with Asians without AMI was greater than the difference between persons with and without AMI in any other group (almost twofold higher overall, and threefold higher in women).

Among adults age ≥25 years with AMI, the prevalence of current smoking was lowest among college graduates (18.7%) (Table 2). By poverty status, prevalence was higher among adults living below the federal poverty level (47.9%) than among those at or above this level (33.3%). By U.S. Census region, prevalence was lowest in the West (31.5%) and Northeast (34.7%) and highest in the Midwest (39.1%) and South (37.8%). By state, the prevalence ranged from 18.2% (Utah) to 48.7% (West Virginia) (Table 3).

## Conclusions and Comments

During 2009–2011, adults with AMI had a high prevalence of cigarette smoking. Sociodemographic variations in the prevalence of current smoking among persons with AMI resembled patterns in the overall population (7,8). Whereas estimates for smoking were reported to be high among persons with AMI, it is likely that these rates would be even higher if the AMI definition included substance use disorders since persons with substance use disorders but no other mental disorder were excluded (9). Increasing awareness of the high smoking prevalence in this population is needed (9). In addition to investing in comprehensive tobacco prevention and control programs at CDC-recommended levels (10), better coordination between tobacco control and mental health programs at the national, state, and community levels is needed. In clinical settings, screening for tobacco use and offering effective cessation treatments, such as medications and counseling, to persons with mental illness†† would likely further reduce tobacco-use prevalence and result in a substantial reduction in tobacco-related morbidity and mortality (10).

The lowest prevalences were observed in the West and the Northeast; by state, the lowest prevalence was observed in Utah. Prevalence was also low in Massachusetts and California, which have achieved successes in reducing smoking in the overall population through implementation of comprehensive tobacco control programs and population-based policy interventions (11). Moreover, Massachusetts substantially reduced smoking prevalence among Medicaid enrollees by establishing and heavily promoting comprehensive Medicaid coverage of evidence-based cessation treatments that minimized cost barriers to their access (12).

In addition to the high prevalence of smoking among persons with AMI, data also indicate that these persons smoke more cigarettes per month and are less likely to have stopped smoking, compared with persons without AMI. There are several possible explanations for these findings. First, because nicotine is a central nervous system stimulant with mood-altering effects, it can temporarily mask negative affect and symptoms associated with mental illness (3). Second, research indicates that other constituents of tobacco smoke can accelerate the metabolism of some mental health medications, thus possibly reducing their effective blood levels (13) and potentially resulting in increased compensatory nicotine intake (13). Third, given that >80% of adult smokers begin smoking during adolescence,§§ those with AMI who smoke also likely started during youth. Factors that might predict the onset of dependence among youths include depressed mood and familiarity with tobacco advertisements (14); adolescents with depressive symptoms might experience increased receptivity to tobacco advertisements, making them more likely to smoke (15). Fourth, the tobacco industry has marketed cigarettes to populations with AMI (16), funded research to show that persons with AMI use nicotine to alleviate negative mood (i.e., self-medicate), provided free or cheap cigarettes to psychiatric facilities, and supported efforts to block smokefree psychiatric hospital policies (3,16). Finally, persons with AMI are uniquely vulnerable. They often lack financial resources, face unstable, stressful living conditions, and have difficulty coping with symptoms of withdrawal; they also might lack health insurance, information on the health effects of smoking, and access to cessation treatments (4,17,18).

**Key Points**

- Cigarette smoking remains the leading preventable cause of disease, disability, and death in the United States. During 2009–2011, nearly 20% of adults reported they had some form of mental illness in the past year, and among those with mental illness, 36% smoked cigarettes.
- About 3 in 10 cigarettes smoked by adults are smoked by those with mental illness.
- Adult smokers with mental illness are less likely to quit than adult smokers without mental illness.
- In addition to sustained and adequately funded comprehensive tobacco control programs, enhanced prevention and cessation efforts among persons with mental illness can further reduce smoking-related death and disease.
- Additional information is available at http://www.cdc.gov/vitalsigns.

Mental health–care providers and facilities have traditionally been reluctant to address tobacco use in their patients (4,17) because of several factors. First, mental health–care providers have been concerned that smoking cessation could interfere with their patients’ treatment (4,17). Some mental health facilities also have used smoking privileges as a reward (4,17). Finally, some mental health–care providers believe that their patients who smoke do not want to or cannot quit (4,17). However, evidence from recent research has suggested that these concerns largely are unfounded; persons with AMI who smoke are as interested in quitting as other smokers, are able to quit successfully, and benefit from evidence-based cessation treatments, although intensive and longer treatment sometimes is required (4,17).

The findings in this report are subject to at least six limitations. First, AMI is an overall measure for DSM-IV disorders and cannot be separated into specific categories, whereas prevalence of smoking can differ among persons with various mental illness diagnoses (2). However, the estimate for the prevalence of AMI reported here is comparable to estimates from other national surveys (6). Second, estimates of smoking were self-reported and not validated by biochemical tests. Although studies of self-reported smoking might yield lower prevalence estimates than studies of serum cotinine (a breakdown product of nicotine) (19), it is unlikely that underreporting would substantially change the estimates reported. The estimates for current smoking in the population overall reported from NSDUH are higher than estimates from other national surveys, such as the National Health Interview Survey (NHIS) (19.0 in 2011), because of variations in the data collection methods and measures used to define current smoking. Nonetheless, both surveys have reported similar trends for current smoking among adults (7,8). Third, this report does not include persons residing in mental health residential communities, for whom smoking practices might differ from persons identified with AMI in the NSDUH sample population. Also, persons in the military were not included, and therefore the findings might not be generalizable to those populations. The report also did not have information about experiences of traumatic stress, which has been shown to be associated with both depressed affect and smoking (20). Fourth, because of small sample sizes, some estimates for American Indians/Alaska Natives were suppressed. Fifth, the data could not be disaggregated for specific Asian subgroups, among whom smoking prevalence is known to vary widely (21). Finally, the estimate that 30.9% of all cigarettes smoked by adults are smoked by those with mental illness is lower than that previously reported (44%) (9), mainly because the estimate in the current study does not include persons who have substance use disorder and no other mental disorder.

The high smoking prevalence among persons with mental illness imposes a heavy burden in lost life expectancy (22) and constitutes a major public health disparity in a uniquely vulnerable population. To reduce this burden and disparity, efforts are needed to raise awareness and increase collaboration among mental health and tobacco control programs at the national, state, and local levels. Several national organizations and federal government agencies have recently called attention to the problem of tobacco use among persons with mental illness. For example, the Substance Abuse and Mental Health Services Administration and the Smoking Cessation Leadership Center have conducted Leadership Academies for Wellness and Smoking Cessation in Behavioral Health to support states in developing action plans to reduce smoking prevalence in this population.¶¶ Implementation of tobacco-free campus policies in mental health facilities and full integration of tobacco dependence treatment into mental health care can contribute to decreasing smoking among persons with AMI. Finally, continued surveillance is needed to track implementation of these policy and clinical interventions and to monitor progress in addressing this disparity.

## Figures and Tables

**TABLE 1:** Percentage of adults with any mental illness, by sex and selected characteristics — National Survey on Drug Use and Health, United States, 2009–2011

	% with any mental illness*					
	
	Men (n=53,700)		Women (n=60,300)		Total (n=114,100)	
			
Characteristic	%	(95% CI)	%	(95% CI)	%	(95% CI)
**Age group (yrs)**						
18–24	24.9	(24.2–25.5)	36.0	(35.3–36.8)	**30.4**	**(29.8–30.9)**
25–44	18.2	(17.4–18.9)	27.2	(26.5–28.0)	**22.8**	**(22.2–23.3)**
45–64	14.0	(13.1–14.9)	20.7	(19.8–21.7)	**17.4**	**(16.8–18.1)**
≥65	8.6	(7.5–9.8)	12.6	(11.5–13.8)	**10.8**	**(10.0–11.7)**
**Race/Ethnicity** †						
White	16.5	(16.0–17.1)	24.5	(24.0–25.1)	**20.7**	**(20.2–21.1)**
Black	15.7	(14.4–17.1)	21.4	(20.1–22.9)	**18.9**	**(17.9–19.9)**
Hispanic	14.1	(13.0–15.3)	20.6	(19.4–21.9)	**17.3**	**(16.5–18.2)**
American Indian/Alaska Native	20.1	(14.4–27.2)	26.3	(21.3–31.9)	**23.4**	**(19.4–28.1)**
Asian	14.9	(12.6–17.6)	16.6	(14.7–18.8)	**15.8**	**(14.3–17.5)**
Other	24.4	(20.3–29.0)	31.1	(27.0–35.5)	**27.8**	**(24.9–30.9)**
**Education** §						
Less than high school graduate	18.3	(16.9–19.9)	22.8	(21.2–24.4)	**20.6**	**(19.5–21.7)**
High school graduate	14.1	(13.2–14.9)	21.1	(20.1–22.2)	**17.7**	**(17.0–18.4)**
Some college	16.3	(15.2–17.5)	23.3	(22.2–24.4)	**20.1**	**(19.3–21.0)**
College graduate	12.6	(11.8–13.4)	20.0	(19.1–20.9)	**16.3**	**(15.7–17.0)**
**Poverty status** ¶						
At or above poverty level	14.8	(14.4–15.3)	21.7	(21.2–22.3)	**18.3**	**(18.0–18.7)**
Below poverty level	26.1	(24.7–27.7)	32.2	(31.0–33.5)	**29.7**	**(28.7–30.8)**
Unknown	25.8	(22.6–29.2)	37.6	(33.6–41.8)	**31.9**	**(29.0–35.0)**
**U.S Census region** **						
Northeast	15.8	(14.8–16.9)	22.9	(21.8–24.0)	**19.5**	**(18.7–20.3)**
Midwest	16.2	(15.4–17.0)	24.3	(23.3–25.2)	**20.4**	**(19.7–21.0)**
South	15.6	(14.9–16.4)	23.2	(22.4–24.0)	**19.6**	**(19.0–20.1)**
West	17.2	(16.1–18.3)	23.2	(22.2–24.4)	**20.3**	**(19.5–21.1)**
**Year**						
2009	15.7	(14.9–16.5)	24.0	(23.1–24.8)	**20.0**	**(19.4–20.6)**
2010	16.8	(16.1–17.7)	23.1	(22.3–24.0)	**20.1**	**(19.5–20.7)**
2011	15.9	(15.1–16.7)	23.0	(22.2–23.9)	**19.6**	**(19.0–20.2)**
**Total**	**16.1**	**(15.7–16.6)**	**23.4**	**(22.9–23.9)**	**19.9**	**(19.5–20.2)**

**Abbreviation:** CI = confidence interval.

* Any mental illness is defined as a diagnosable mental, behavioral, or emotional disorder, other than a developmental or substance use disorder, that met the criteria found in the 4th edition of the “Diagnostic and Statistical Manual of Mental Disorders (DSM-IV).” For details on the methodology, see Section B.4.3 in Appendix B of the Results from the 2011 National Survey on Drug Use and Health: Mental Health Findings.

† Persons identified as Hispanic might be of any race. Persons identified as white, black, Asian, American Indian/Alaska Native, or Other are all non-Hispanic. The five racial/ethnic categories are mutually exclusive. Other includes Native Hawaiians or Other Pacific Islanders and persons of two or more races.

§ Among adults aged ≥25 years.

¶ Based on reported family income and poverty thresholds published by the U.S. Census Bureau.

** Northeast: Connecticut, Maine, Massachusetts, New Hampshire, New Jersey, New York, Pennsylvania, Rhode Island, and Vermont. Midwest: Illinois, Indiana, Iowa, Kansas, Michigan, Minnesota, Missouri, Nebraska, North Dakota, Ohio, South Dakota, and Wisconsin. South: Alabama, Arkansas, Delaware, District of Columbia, Florida, Georgia, Kentucky, Louisiana, Maryland, Mississippi, North Carolina, Oklahoma, South Carolina, Tennessee, Texas, Virginia, and West Virginia. West: Alaska, Arizona, California, Colorado, Hawaii, Idaho, Montana, Nevada, New Mexico, Oregon, Utah, Washington, and Wyoming.

**TABLE 2:** Percentage of adults who smoke cigarettes,* by mental illness status,† sex, and selected characteristics — National Survey on Drug Use and Health, United States, 2009–2011

	% of persons with any mental illness who smoke cigarettes						% of persons with no mental illness who smoke cigarettes					
		
	Men (n=11,100)		Women (n=18,300)		Total (n=29,400)		Men (n=42,700)		Women (n=42,000)		Total (n=84,700)	
						
Characteristic	%	(95% CI)	%	(95% CI)	%	(95% CI)	%	(95% CI)	%	(95% CI)	%	(95% CI)
**Age group (yrs)**												
18–24	45.2	(43.6–46.9)	39.1	(37.9–40.3)	**41.6**	**(40.6–42.6)**	36.7	(35.7–37.6)	24.9	(24.1–25.7)	**31.3**	**(30.6–32.0)**
25–44	44.7	(42.5–46.9)	37.8	(36.2–39.5)	**40.5**	**(39.3–41.8)**	29.8	(28.8–30.8)	21.7	(20.9–22.6)	**25.9**	**(25.2–26.6)**
45–64	34.7	(31.6–37.9)	32.8	(30.5–35.1)	**33.5**	**(31.7–35.4)**	22.1	(21.0–23.3)	19.2	(18.2–20.3)	**20.7**	**(19.9–21.5)**
≥65	18.3	(13.0–25.3)	10.1	(7.5–13.5)	**13.0**	**(10.3–16.1)**	9.1	(7.8–10.5)	8.6	(7.6–9.7)	**8.8**	**(8.0–9.7)**
**Race/Ethnicity** §												
White	40.4	(38.7–42.1)	36.0	(34.8–37.2)	**37.7**	**(36.7–38.7)**	24.4	(23.7–25.1)	20.1	(19.4–20.7)	**22.3**	**(21.7–22.8)**
Black	41.5	(37.1–46.1)	29.5	(26.5–32.6)	**34.0**	**(31.5–36.5)**	25.9	(24.1–27.9)	19.2	(17.5–20.9)	**22.3**	**(21.0–23.7)**
Hispanic	38.2	(34.0–42.6)	26.8	(24.1–29.8)	**31.6**	**(29.1–34.2)**	25.5	(23.9–27.2)	13.4	(12.1–14.7)	**19.8**	**(18.7–20.9)**
American Indian/Alaska Native	—¶	—¶	56.0	(44.9–66.5)	**54.7**	**(45.3–63.7)**	35.0	(27.9–42.9)	26.3	(20.7–32.9)	**30.5**	**(25.7–35.7)**
Asian	26.6	(20.3–34.1)	16.0	(12.4–20.4)	**20.6**	**(17.2–24.6)**	15.9	(13.6–18.5)	5.5	(4.2–7.3)	**10.4**	**(9.0–11.9)**
Other	35.8	(27.3–45.3)	43.1	(36.4–50.0)	**40.0**	**(34.5–45.7)**	26.3	(22.4–30.7)	26.3	(21.8–31.4)	**26.3**	**(23.2–29.6)**
**Education** **												
Less than high school graduate	53.0	(48.5–57.4)	41.5	(37.8–45.3)	**46.6**	**(43.6–49.6)**	34.8	(32.8–36.9)	22.7	(20.9–24.7)	**28.9**	**(27.6–30.3)**
High school graduate	42.8	(39.5–46.3)	38.6	(36.1–41.2)	**40.2**	**(38.2–42.3)**	28.4	(27.2–29.7)	21.9	(20.8–23.0)	**25.2**	**(24.3–26.0)**
Some college	39.3	(35.9–42.9)	37.5	(35.2–39.8)	**38.1**	**(36.2–40.2)**	23.5	(22.2–24.9)	19.9	(18.7–21.1)	**21.6**	**(20.7–22.5)**
College graduate	22.0	(19.4–24.9)	16.7	(14.9–18.6)	**18.7**	**(17.2–20.3)**	11.7	(10.8–12.6)	9.5	(8.7–10.4)	**10.6**	**(10.0–11.3)**
**Poverty status** ††												
At or above poverty level	36.8	(35.2–38.5)	30.9	(29.8–32.0)	**33.3**	**(32.3–34.2)**	22.9	(22.3–23.5)	16.8	(16.3–17.4)	**20.0**	**(19.5–20.4)**
Below poverty level	52.8	(49.4–56.2)	45.1	(42.8–47.4)	**47.9**	**(45.9–49.8)**	38.3	(36.3–40.4)	28.6	(26.9–30.3)	**32.8**	**(31.5–34.1)**
Unknown	24.9	(19.2–31.6)	23.8	(18.8–29.6)	**24.2**	**(20.6–28.2)**	21.4	(16.3–27.5)	17.4	(14.0–21.4)	**19.5**	**(16.2–23.3)**
**U.S Census region** §§												
Northeast	37.6	(34.4–40.8)	32.9	(30.6–35.4)	**34.7**	**(32.8–36.7)**	22.9	(21.6–24.3)	18.8	(17.7–20.1)	**20.9**	**(20.0–21.8)**
Midwest	42.9	(40.4–45.4)	36.7	(34.7–38.7)	**39.1**	**(37.5–40.7)**	25.8	(24.7–26.9)	20.8	(19.8–21.9)	**23.4**	**(22.6–24.2)**
South	41.9	(39.3–44.5)	35.3	(33.5–37.1)	**37.8**	**(36.3–39.3)**	26.1	(25.0–27.2)	19.2	(18.2–20.2)	**22.7**	**(21.9–23.5)**
West	35.1	(32.1–38.2)	29.0	(27.0–31.0)	**31.5**	**(29.7–33.3)**	21.6	(20.3–23.0)	14.4	(13.3–15.5)	**18.1**	**(17.2–19.0)**
**Year**												
2009	41.3	(38.9–43.8)	34.1	(32.3–35.9)	**36.8**	**(35.3–38.4)**	24.5	(23.4–25.5)	19.2	(18.3–20.2)	**21.9**	**(21.2–22.6)**
2010	40.2	(37.8–42.6)	34.2	(32.4–36.1)	**36.6**	**(35.2–38.1)**	24.7	(23.7–25.7)	18.3	(17.3–19.3)	**21.5**	**(20.8–22.3)**
2011	37.4	(35.0–39.9)	33.0	(31.2–34.8)	**34.7**	**(33.3–36.2)**	24.1	(23.1–25.1)	17.7	(16.8–18.6)	**20.9**	**(20.2–21.6)**
**Total**	**39.6**	**(38.2–41.1)**	**33.8**	**(32.7–34.8)**	**36.1**	**(35.2–36.9)**	**24.4**	**(23.8–25.0)**	**18.4**	**(17.8–18.9)**	**21.4**	**(21.0–21.9)**

**Abbreviation:** CI = confidence interval.

* Persons who reported ever smoking all or part of a cigarette in the 30 days preceding the interview.

† Any mental illness is defined as a diagnosable mental, behavioral, or emotional disorder, other than a developmental or substance use disorder, that met the criteria found in the 4th edition of the “Diagnostic and Statistical Manual of Mental Disorders (DSM-IV).” For details on the methodology, see Section B.4.3 in Appendix B of the Results from the 2011 National Survey on Drug Use and Health: Mental Health Findings.

§ Persons identified as Hispanic might be of any race. Persons identified as white, black, Asian, American Indian/Alaska Native, or Other are all non-Hispanic. The five racial/ethnic categories are mutually exclusive. Other includes Native Hawaiians or Other Pacific Islanders and persons of two or more races.

¶ No estimate reported because of low precision.

** Among adults aged ≥25 years.

†† Based on reported family income and poverty thresholds published by the U.S. Census Bureau.

§§ Northeast: Connecticut, Maine, Massachusetts, New Hampshire, New Jersey, New York, Pennsylvania, Rhode Island, and Vermont. Midwest: Illinois, Indiana, Iowa, Kansas, Michigan, Minnesota, Missouri, Nebraska, North Dakota, Ohio, South Dakota, and Wisconsin. South: Alabama, Arkansas, Delaware, District of Columbia, Florida, Georgia, Kentucky, Louisiana, Maryland, Mississippi, North Carolina, Oklahoma, South Carolina, Tennessee, Texas, Virginia, and West Virginia. West: Alaska, Arizona, California, Colorado, Hawaii, Idaho, Montana, Nevada, New Mexico, Oregon, Utah, Washington, and Wyoming.

**TABLE 3:** Percentage of adults who smoke cigarettes, by mental illness status and state — National Survey on Drug Use and Health, United States, 2009–2011

	% with any mental illness*		% of persons with any mental illness who smoke cigarettes†		% of persons with no mental illness who smoke cigarettes†	
			
State	%	(95% CI)	%	(95% CI)	%	(95% CI)
**United States overall**	**19.9**	**(19.5–20.2)**	**36.1**	**(35.2–36.9)**	**21.4**	**(21.0–21.9)**
**State median**	**20.4**		**36.7**		**22.1**	
Alabama	23.8	(21.0–26.9)	47.7	(41.3–54.1)	22.5	(19.5–25.8)
Alaska	20.5	(17.6–23.7)	40.1	(33.4–47.1)	28.8	(24.3–33.8)
Arizona	20.1	(17.3–23.3)	39.7	(33.1–46.7)	20.1	(16.9–23.6)
Arkansas	22.5	(20.1–25.2)	41.0	(34.7–47.6)	25.5	(22.4–28.8)
California	18.7	(17.5–20.0)	30.0	(27.2–32.9)	15.8	(14.6–17.2)
Colorado	19.9	(17.1–23.0)	31.4	(25.4–38.0)	20.3	(16.9–24.2)
Connecticut	17.8	(14.7–21.2)	35.7	(27.7–44.6)	20.4	(17.1–24.2)
Delaware	20.1	(17.3–23.1)	34.9	(28.6–41.9)	24.3	(21.1–27.9)
District of Columbia	21.3	(18.4–24.5)	34.6	(27.1–42.9)	20.8	(17.4–24.6)
Florida	17.8	(16.5–19.2)	36.6	(32.8–40.6)	21.2	(19.8–22.8)
Georgia	17.2	(15.2–19.3)	24.0	(18.8–30.1)	20.0	(17.2–23.2)
Hawaii	20.4	(17.7–23.2)	29.7	(24.4–35.6)	20.3	(17.1–23.9)
Idaho	27.2	(24.7–29.9)	35.5	(29.8–41.6)	21.3	(18.2–24.8)
Illinois	18.3	(17.1–19.5)	38.0	(34.5–41.6)	22.1	(20.5–23.8)
Indiana	22.3	(19.5–25.3)	38.8	(33.3–44.6)	24.4	(20.5–28.9)
Iowa	20.7	(18.0–23.6)	41.2	(33.6–49.4)	22.4	(19.3–25.9)
Kansas	18.3	(16.0–20.8)	37.5	(30.6–45.0)	23.6	(20.3–27.2)
Kentucky	21.2	(18.4–24.3)	41.8	(36.0–47.9)	31.8	(27.6–36.2)
Louisiana	20.7	(18.3–23.4)	44.9	(36.3–53.8)	25.5	(21.7–29.7)
Maine	18.4	(15.9–21.1)	35.5	(29.6–41.8)	25.2	(22.1–28.5)
Maryland	19.4	(16.7–22.4)	27.7	(23.1–32.8)	18.5	(15.1–22.6)
Massachusetts	19.3	(16.9–22.0)	29.7	(23.3–37.0)	16.8	(14.2–19.9)
Michigan	21.6	(20.3–23.0)	41.5	(38.1–45.0)	24.6	(23.0–26.2)
Minnesota	19.0	(16.6–21.7)	40.2	(33.6–47.1)	19.9	(17.6–22.5)
Mississippi	21.8	(19.4–24.4)	39.9	(33.6–46.5)	25.3	(22.6–28.2)
Missouri	20.3	(18.0–22.8)	39.4	(33.2–46.1)	26.1	(23.0–29.5)
Montana	22.4	(20.1–24.8)	30.9	(24.9–37.7)	24.4	(21.3–27.7)
Nebraska	19.1	(16.5–21.9)	38.5	(31.9–45.4)	22.2	(18.9–26.0)
Nevada	20.7	(17.5–24.2)	36.0	(28.4–44.4)	22.8	(19.1–26.9)
New Hampshire	20.9	(18.1–24.0)	37.5	(31.4–44.1)	20.3	(17.5–23.4)
New Jersey	17.4	(15.0–20.1)	35.6	(30.0–41.6)	21.6	(18.7–24.9)
New Mexico	19.9	(17.3–22.8)	34.8	(28.8–41.4)	19.9	(16.7–23.5)
New York	20.4	(19.1–21.7)	33.0	(29.8–36.4)	20.2	(18.8–21.7)
North Carolina	18.3	(16.4–20.4)	41.8	(35.0–48.9)	22.5	(19.7–25.7)
North Dakota	17.9	(15.6–20.6)	35.6	(30.2–41.4)	21.2	(19.1–23.6)
Ohio	22.2	(20.8–23.7)	39.0	(35.6–42.5)	25.1	(23.5–26.8)
Oklahoma	22.2	(19.5–25.1)	45.5	(38.7–52.5)	28.9	(24.9–33.3)
Oregon	21.1	(18.3–24.3)	35.3	(29.3–41.8)	21.2	(18.4–24.3)
Pennsylvania	19.5	(18.3–20.9)	38.8	(35.2–42.6)	23.2	(21.4–25.1)
Rhode Island	23.9	(20.1–28.2)	34.1	(27.5–41.4)	21.9	(18.0–26.5)
South Carolina	21.0	(18.3–23.9)	43.3	(35.9–50.9)	27.0	(23.5–30.9)
South Dakota	18.1	(15.1–21.5)	40.8	(33.1–48.9)	23.2	(19.8–26.8)
Tennessee	25.8	(22.9–28.8)	45.0	(37.7–52.5)	25.6	(21.7–29.9)
Texas	17.5	(16.3–18.7)	33.9	(30.7–37.3)	21.3	(19.7–23.0)
Utah	26.9	(24.2–29.8)	18.2	(14.3–22.9)	12.3	(9.7–15.5)
Vermont	22.2	(19.5–25.0)	38.0	(32.5–43.8)	19.9	(16.9–23.2)
Virginia	20.3	(18.2–22.5)	35.1	(29.5–41.1)	19.7	(16.0–23.9)
Washington	23.9	(21.3–26.8)	31.1	(25.6–37.2)	21.1	(17.8–24.8)
West Virginia	23.5	(20.8–26.3)	48.7	(40.7–56.8)	29.1	(24.9–33.8)
Wisconsin	20.0	(17.2–23.2)	35.6	(29.1–42.6)	20.9	(17.4–25.0)
Wyoming	21.8	(19.2–24.7)	36.7	(30.3–43.6)	22.8	(19.5–26.5)

**Abbreviation:** CI = confidence interval.

* Any mental illness is defined as a diagnosable mental, behavioral, or emotional disorder, other than a developmental or substance use disorder that met the criteria found in the 4th edition of the “Diagnostic and Statistical Manual of Mental Disorders (DSM-IV).” For details on the methodology, see Section B.4.3 in Appendix B of the Results from the 2011 National Survey on Drug Use and Health: Mental Health Findings.

† Persons who reported ever smoking part or all of a cigarette, and who, at the time of interview, reported smoking part or all of a cigarette within the preceding 30 days.

## Footnotes

* Additional information available at http://www.cdc.gov/tobacco/data_statistics/fact_sheets/fast_facts/index.htm.

† Additional information available at http://www.who.int/tobacco/mpower/mpower_report_full_2008.pdf.

§ Additional information available at http://healthypeople.gov/2020/topicsobjectives2020.

¶ Additional information available at http://www.samhsa.gov/data/NSDUH/2k11MH_FindingsandDetTables/Index.aspx.

** Additional information available at: http://www.samhsa.gov/data/2k13/NSDUH093/sr093-smoking-mental-illness.pdf.

†† Additional information available at http://www.ahrq.gov/clinic/tobacco/treating_tobacco_use08.pdf.

§§ Additional information available at http://www.surgeongeneral.gov/library/reports/preventing-youth-tobacco-use/full-report.pdf.

¶¶ Additional information available at http://smokingcessationleadership.ucsf.edu/LeadershipAcademies.htm.
